# Facile route to freestanding CH_3_NH_3_PbI_3_ crystals using inverse solubility

**DOI:** 10.1038/srep11654

**Published:** 2015-06-30

**Authors:** Jeannette M. Kadro, Kazuteru Nonomura, David Gachet, Michael Grätzel, Anders Hagfeldt

**Affiliations:** 1 Laboratory for Photomolecular Science, Institute of Chemical Sciences and Engineering, École Polytechnique Fédérale de Lausanne, CH-1015 Lausanne, Switzerland; 2 Attolight AG, EPFL Innovation Park, Bâtiment D, CH-1015 Lausanne, Switzerland; 3 Laboratory for Photonics and Interfaces, Institute of Chemical Sciences and Engineering, École Polytechnique Fédérale de Lausanne, CH-1015 Lausanne, Switzerland

## Abstract

CH_3_NH_3_PbI_3_ was found to exhibit inverse solubility at high temperatures in γ-butyrolactone. Making use of this unusual, so far unreported phenomenon, we present a facile method for the growth of freestanding crystals of CH_3_NH_3_PbI_3_ from solution without addition of any capping agents or seed particles. Large, strongly faceted crystals could be grown within minutes. This finding may aid in understanding the crystallization process of CH_3_NH_3_PbI_3_ from solution that may lead to improved morphological control of film deposition for a range of device architectures. Our process offers a facile and rapid route to freestanding crystals for use in a broad range of characterization techniques.

Since the first peer-reviewed publications of metalorgano halide perovskite based solar cells in 2009^1^, research in materials of this family has undergone immense revival. They unite a broad range of suitable characteristics including long-range charge diffusion^2^, high extinction coefficient as well as ease of fabrication^3^. With solar-to-energy conversion efficiencies now exceeding 20%^4^, some already regard them as possible competitors to the well-established silicon technology^5^,^6^. Despite the undisputable impressive developments in conversion efficiencies for laboratory scale devices since the first reports, many non-trivial issues including long-term device stability and reproducibility, material degradation and toxicity of the water-soluble lead halide as well the establishment of reliable device testing protocols remain yet to be solved^7^. Much effort has been invested into the optimization of deposition conditions for thin films for application in solar cells with processes ranging from simple one-step solution deposition over sequential deposition processes^8^,^9^ to vapor based processes^10^,^11^.

In most reported solution growth methods for freestanding crystals, supersaturation is achieved by cooling saturated solutions or by addition of poor solvents^12^,^13^. To the best of our knowledge, none of the reported protocols for the formation of CH_3_NH_3_PbI_3_ structures, including thin films as well as freestanding products, rely on inverse solubility. We report a facile method for rapid growth of large, freestanding crystals of CH_3_NH_3_PbI_3_ from a solution of γ-butyrolactone (GBL) through heating at unconventionally high temperatures. To our knowledge, the possibility of formation of crystalline CH_3_NH_3_PbI_3_ from solution in a good solvent without additives has not been reported.

Several methods for the growth of freestanding crystals of CH_3_NH_3_PbI_3_ from solution have been reported^14^. Commonly, growing large freestanding crystals in solution is a slow process but Yang *et al.*^15^ have demonstrated a simple and efficient method for growth of CH_3_NH_3_PbI_3_ via sequential solution growth method. Their process allowed for the formation of crystals of a range of habits and sizes. Recently, Kollek *et al.* have reported a novel single-precursor route for the preparation of a range of nanostructures including porous, shape-anisotropic single crystals via crystal-to-crystal transformation^16^.

Studies of important physical properties such as thermal conductivity, Hall Effect and others, commonly have to rely on freestanding structures rather than thin films^17^. Reported formations of CH_3_NH_3_PbX_3_ (x = Cl, Br, I) include nanowires^18^, size-controlled cuboids on mesoporous substrates^19^, freestanding crystals^12^,^13^,^15^ and freestanding nanoparticles^16^,^20^.

Our findings show that crystal growth can be achieved through heating, rather than cooling, a concentrated solution containing only two precursor salts, indicating negative temperature dependence of the solubility of CH_3_NH_3_PbI_3_. The growth solution composition we use to grow freestanding crystals has been commonly used for spin coating CH_3_NH_3_PbI_3_ to fabricate crystalline thin films as components in solar cell devices^21^,^22^,^23^.

## Results and Discussion

PbI_2_ and CH_3_NH_3_I were used as precursors and γ-butyrolactone (GBL) was used as solvent. No additives such as capping agents, stabilizers or seeding particles were used. Both salts were dissolved in GBL at 1:1 molar ratio. The powders were dissolved under vigorous stirring on a hotplate set at 100 °C until clear solutions were obtained. To grow crystals, the temperature of the hotplate was rapidly increased to 190 °C and held at that temperature until formation of crystals of desired dimensions was achieved. For concentrations stable at room temperature, crystals were found to start forming when the solution temperature exceeded 135 °C. Detailed experimental procedures are available in the methods section.

Large crystals of 1 mm length could be grown within minutes at precursor concentrations of 0.905 M CH_3_NH_3_PbI_3_. Crystal formation occurred in solutions with equimolar ratio of the two precursor salts as well as for solutions with as much as 70% excess of CH_3_NH_3_I. No crystal growth occurred in solutions with higher excess of CH_3_NH_3_I. Addition of excess quantities of CH_3_NH_3_I was found to lead to the formation of large numbers of small crystals while addition of excess amounts of PbI_2_ aided the formation of small numbers of larger crystals.

**Figure 1:**
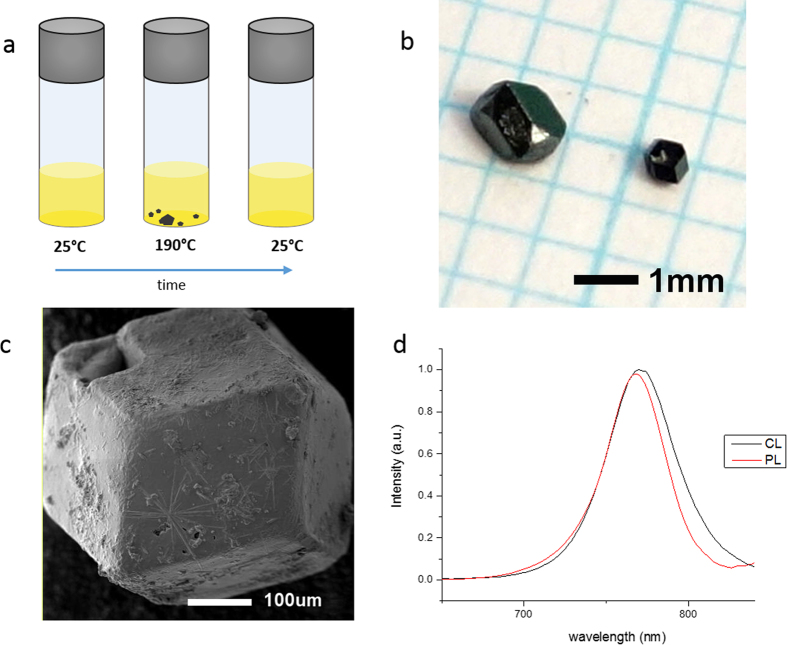
**a**) schematic experimental observation **b**) photograph of freshly grown crystals, 1 blue square = 1 mm^2^ **c**) Secondary electron image of CH_3_NH_3_PbI_3_ crystal **d**) PL and CL spectra of CH_3_NH_3_PbI_3_.

The process is outlined schematically in Fig. 1a. Crystals were recovered from the hot growth solutions and dried carefully before storage in anhydrous chlorobenzene, hexane or dichloromethane. Upon removal of the growth vessel from the hot plate, the crystals dissolve rapidly in the solution upon cooling. Small crystals are easily dissolved upon removal from the hot growth solution as the liquid film remaining on their surface cools down rapidly and supersaturation at the interface is no longer maintained. Trials replacing GBL partially or completely with *N, N-*dimethylformamide (DMF) or dimethylsulfoxide (DMSO) as well as partial halide substitution by chloride or bromide were unsuccessful to date.

Figure 1b shows a photograph of CH_3_NH_3_PbI_3_ crystals freshly grown from a 0.9 M equimolar precursor solution within 25 min. The surface of the freshly grown crystals is black and glossy and appears smooth to the eye. After exposure to ambient air, degradation leads to the formation of a yellow phase on the surfaces within days^13^,^24^.

All crystals were strongly faceted but varied in habit. Crystals mostly formed as rhombic dodecahedra or rhombo-hexagonal dodecahedra. Such faceting is consistent with the *I4/mcm* space group reported for this material grown from different routes^24^.

The micrograph in Fig. 1c shows a typical crystal of ca. 500 μm diameter grown from 0.9 M precursor solution. Elongated needle-like structures on the surface indicate the beginning of degradation due to exposure to humidity during storage. The surface of the crystal is not entirely smooth and may have been attacked by the storage solvent.

Figure 1d shows photoluminescence (PL) and cathodoluminescence (CL) spectra of CH_3_NH_3_PbI_3_ grown with our process. PL spectra were collected on finely ground particles suspended in chloroform and CL spectra on a single point on the surface of the crystal in Fig. 1c. PL spectra show a single emission peak at 775 nm after excitation at 480 nm. Peaks for both PL and CL almost coincide and are well within the range of expected values for the reported band gap ranging around 1.5 eV of tetragonal CH_3_NH_3_PbI_3_^14^,^15^.

Thermogravimetric analysis (TGA) carried out between 30 °C and 220 °C under continuous Argon flow on a number of unground crystals freshly prepared revealed a 0.6% weight loss between 30 °C and 65 °C, most likely stemming from evaporation of DCM which was used as a protective storage medium (Figure S3).

**Figure 2:**
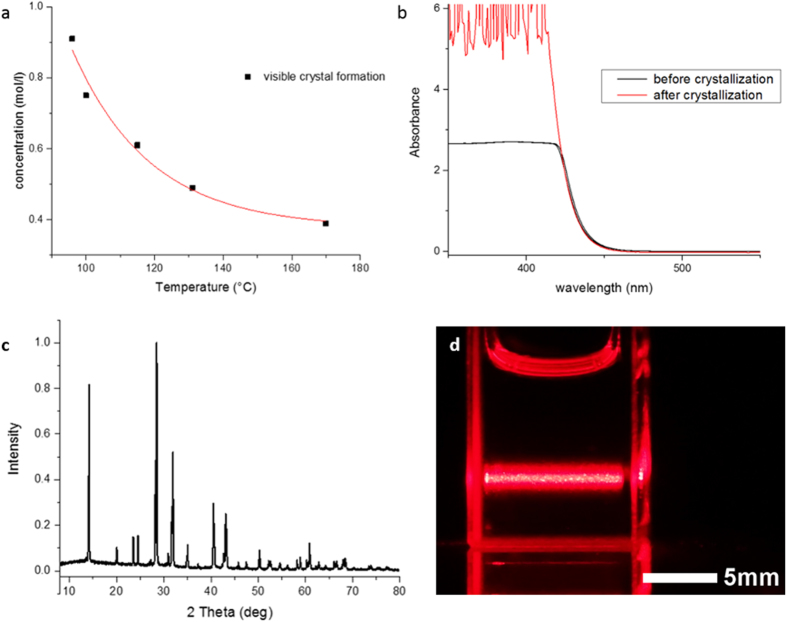
**a**) solubility curve for equimolar solutions of CH_3_NH_3_PbI_3_ in GBL **b**) room temperature UV-vis absorption spectra of growth solutions 0.9 M before and after crystal growth and dissolution **c**) powder XRD pattern on fine powder of ground crystals **d**) Tyndall effect on growth solution.

To assess the crystallinity of the material further, XRD patterns were collected on a finely ground powder prepared by the same process (Fig. 2). CH_3_NH_3_PbI_3_ has a cubic *Pm*
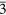
*m* high temperature phase with phase transition from its *I4mcm* tetragonal room temperature phase occurring around 56 °C at atmospheric pressure^24^. Whether the crystals form as pseudo cubic crystals in the hot solution and then undergo a phase transition to the tetragonal phase after cooling to room temperature remains to be investigated.

Commonly, crystals of this type of material are grown by cooling solutions to achieve supersaturation^13^,^14^. Methods recently reported relying on a sequential approach to the formation of CH_3_NH_3_PbI_3_ freestanding crystals through controlled addition of PbI_2_ crystals to a solution of CH_3_NH_3_I or anti-solvent vapor based methods rely on decreased solubility of the target material in the growth medium^15^,^25^. To the best of our knowledge, neither CH_3_NH_3_PbI_3_, nor other metalorgano halide perovskites are known to be able to crystallize in their own solvents at elevated temperatures. Such crystallization requires negative temperature dependence of the solubility. Figure 2a shows the temperature dependence of the onset of crystallization in equimolar precursors.

Broadly speaking, nucleation in supersaturated solutions can be divided into homogeneous and heterogeneous nucleation^26^,^27^. For our case, the nature of the nucleation remains under investigation. When fast heating rates were employed, crystals were found to form at the bottom of the growth vessel while when the temperature was raised slowly (<5 °C/min) they appeared to be forming floating in the bulk of the solution and only sink to the bottom of the vial after reaching a certain critical size. We thus assume that crystal formation at the bottom of the growth vessel is not primarily due to crystals forming at surface flaws of the vessel but that in fast heating regimes nucleation temperature is only attained in a small solution volume close to the heat source. Slow heating regimes lead to a more uniform distribution of temperature in the growth solution. As the heat is conveyed to the growth vessel from a hotplate below, we assume large temperature differences in the growth solution.

Figure 2b shows optical absorption spectra of 0.9 M solutions collected before and after crystallization and dissolution as schematically shown in Fig. 1a. Shape and onset of the spectra are practically identical, demonstrating the reversibility of the process. The higher absorption of the solution after crystallization and dissolution of the crystals likely stems from a slight increase of concentration due to solvent loss under the thermal treatment.

As the experiments were performed in ambient conditions it has to be assumed that the hygroscopic solvent was not water-free. At high temperatures the liquid was subjected to lead to small bubbles escaping from the solution which in turn may induce cavity nucleation. To minimize the effect of collapsing bubbles stemming from boiling water content, freshly distilled GBL was used to grow crystals in a nitrogen glovebox under water-free conditions; this was not found to affect the crystallization temperature. Cavitation induced nucleation cannot be ruled out to be a contributing process for this solution based growth considering the temperature range used, but the observation that crystallization is not delayed in conditions where the effect should be minimal indicates that it is likely not the primary nucleation process taking place.

Growth precursors were found to exhibit Tyndall effects when a red laser beam passed through the solution as it was heated to induce crystal formation, shown in Fig. 2. The effect could clearly be observed at all temperatures from 25 °C to 140 °C, confirming that particles are present at all stages. At least a portion of the particles are large enough to easily be seen by the unaided eye as the beam passes through the liquid. The term “solution” should therefore be used with caution when referring to this type of CH_3_NH_3_PbI_3_ precursor as it is apparent that they actually constitute of a dispersant in a solution. Similar observations have been reported by Yan *et al.*, attributing the dispersant to the formation of soft colloidal frameworks^28^.

Precursors filtered through 0.05 μm PTFE syringe filters did no longer produce crystals upon heating unless the initial concentration was above 1M. As a consequence, homogeneous nucleation can be ruled out.

Remarkably, without exception, the material recovered from this process was found to form strongly faceted crystals rather than shapeless formations. Even fast heating regimes of >10 °C/min resulted in formation of strongly faceted crystals. Larger crystals typically exhibit strong distortion as result of preferential growth of favored facets. Optimized experimental geometry may enable growth of undistorted large crystals. The observation that the facets of many of the crystals recovered from the solution were found to correspond to the *I4mcm* space group indicates the possibility of using this high temperature growth process for the preparation of single crystals.

## Conclusion

CH_3_NH_3_PbI_3_ was found to exhibit negative temperature dependence of solubility in a common organic solvent. Using this rare phenomenon, we have presented a facile method for the preparation of large, freestanding crystals of CH_3_NH_3_PbI_3_ without relying on capping agents and other additives. Crystals could be grown without employing particular equipment in very short growth times. The nature of the nucleation process as well as the kinetic processes enabling crystal growth under the investigated conditions as well as the reasons for the observed extraordinary phenomenon of inverse solubility of CH_3_NH_3_PbI_3_ remain under investigation. Our finding may further aid in understanding the mechanism behind the crystallization of CH_3_NH_3_PbI_3_ which is crucial for the optimization of deposition processes. Better understanding of the crystallization process as well as quantification of the solubility at high temperatures will facilitate the development of new deposition techniques of CH_3_NH_3_PbI_3_ on flat or structured substrates for a range of applications.

## Methods

### Crystal growth

The growth solutions were prepared under nitrogen by dissolving PbI_2_ (99%, Sigma Aldrich) and CH_3_NH_3_I in γ-Butyrolactone (98%, Sigma Aldrich) in 1:1 molar ratio. Commercially available chemicals were used as received without further purification. Solutions of 0.3 M to 0.9 M were prepared. The solvent was added to the salts at room temperature. Tightly closed vials were heated on a conventional hotplate at 100 °C until no visible traces of undissolved salts remained. Solutions were then transferred out from the glovebox. Once the salts were completely dissolved, no visible turbidity or precipitation occurred for days for equimolar solutions and for solutions with excess of CH_3_NH_3_I. Solutions below 0.8 M concentration were found to be stable for weeks.

To grow the crystals, screw-top scintillation vials of 15 ml volume were used for solution volumes of 1.5 ml. After stabilizing the temperature of the solutions for 10 min at 100 °C, temperature of the hotplate was increased until black crystals formed at around 190 °C. To avoid boilover, the solution temperature was monitored by directly inserting a thermometer into a vial of identical dimensions with the corresponding volume of the pure solvent.

Crystals were recovered from the hot solutions and immediately dried carefully prior to storage.

### Material Characterization

Powder X-ray diffraction patterns were collected on a Bruker D8 Advance X-ray diffractometer using Cu Kα radiation. Small crystals were prepared by rapid heating in order to obtain a powder fine enough for powder diffraction.

Photoluminescence spectra were collected on a Horiba Jobin Yvon Fluorolog-3 spectrofluorometer with excitation at 480 nm.

The CL-SEM measurements were performed at room temperature using an Attolight Rosa 4634 CL microscope, with a beam probe of 5 nm, an accelerating voltage of 2 kV and a beam current of 20nA. This CL microscope tightly integrates a high numerical aperture (NA 0.72) achromatic reflective lens within the objective lens of a field emission gun scanning electron microscope (FEG-SEM). The focal plane of the light lens matches the FEG-SEM optimum working distance. The CL collection efficiency is constant over a 300 μm field of view so that CL emission can be compared quantitatively between two separate points. CL was spectrally resolved with a Czerny-Turner spectrometer (320 mm focal length, 150 grooves/mm grating) and measured with an UV-vis. CCD camera.

Optical absorption spectra were collected on a Varian Cary 5 spectrophotometer.

Thermogravimetric analysis (TGA) was carried out on a Perkin Elmer Pyris 6 TGA Thermogravimetric Analyzer at heating and cooling rates of 1 K/min under constant Argon flow of 20 ml/min.

## Additional Information

**How to cite this article**: Kadro, J. M. *et al.* Facile route to freestanding CH_3_NH_3_PbI_3_ crystals using inverse solubility. *Sci. Rep.* **5**, 11654; doi: 10.1038/srep11654 (2015).

## Supplementary Material

Supplementary Information
