# Supplementary material for: Facile route to freestanding CH3NH3PbI3 crystals using inverse solubility
Source: Sci Rep. 2015 Jun 30;5:11654. doi: 10.1038/srep11654 (PMC4650687; doi:10.1038/srep11654)
Supplement: Supplementary Information [file srep11654-s1.pdf]

Facile route to freestanding $CH_3NH_3PbI_3$ crystals using inverse solubility

Supporting Information

Jeannette M. Kadro[1], Kazuteru Nonomura[1], David Gachet[2], Michael Grätzel[3], Anders Hagfeldt[1*]

1 Laboratory for Photomolecular Science, Institute of Chemical Sciences and Engineering, École Polytechnique Fédérale de Lausanne, CH-1015 Lausanne, Switzerland

2 Attolight AG, EPFL Innovation Park, Bâtiment D, CH-1015 Lausanne, Switzerland

3 Laboratory for photonics and interfaces, Institute of Chemical Sciences and Engineering, École Polytechnique Fédérale de Lausanne, CH-1015 Lausanne, Switzerland

*corresponding author: anders.hagfeldt@epfl.ch

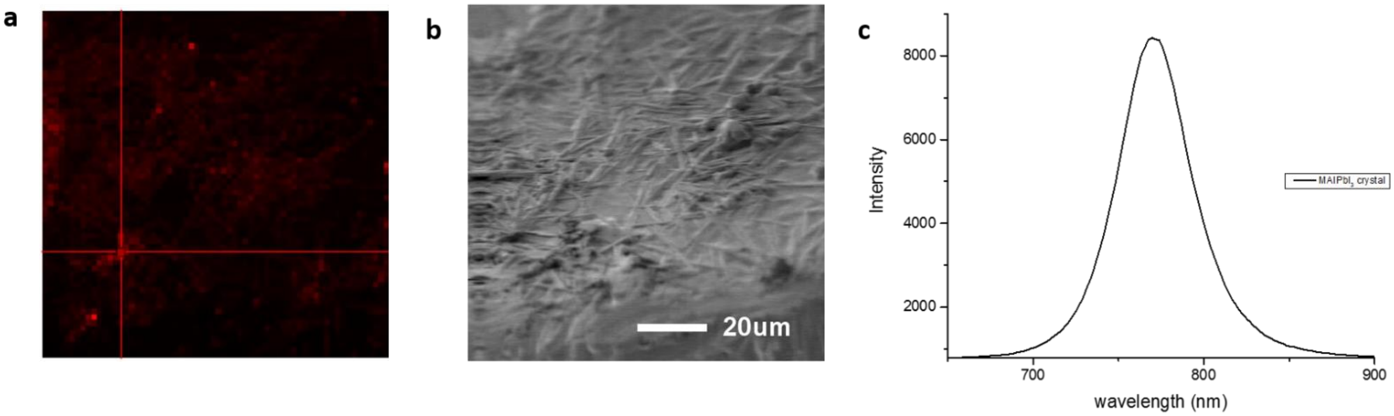

Figure S1 Hyperspectral map of crystal surface b) Secondary electron image of the surface area in a) and c) CL spectrum on single pixel

Figure S1a shows a hyperspectral map collected on the surface of the crystal shown in Figure 1c. The red channel was assigned to a band of 60nm width with central wavelength at 765nm. The needle-like formations apparent on the secondary electron image in b) indicated onset of surface degradation, c) shows CL spectrum extracted from a single pixel on the same sample corresponding to a).

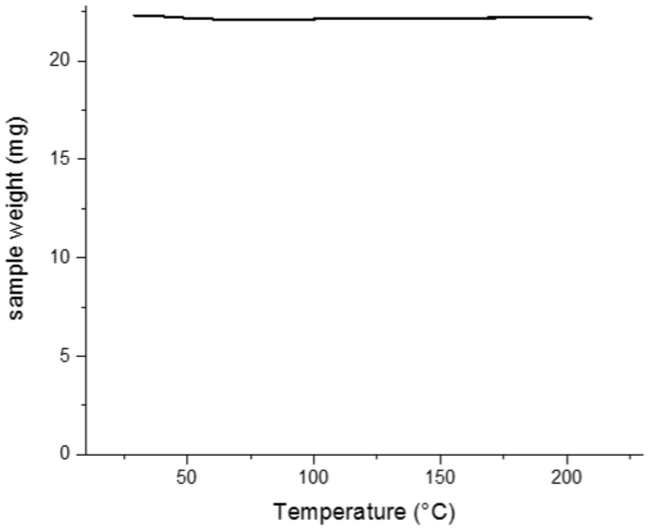

Figure S2 TGA on unground crystals

Thermogravimetric analysis was carried out on a Perkin Elmer Pyris 6 TGA Thermogravimetric Analyzer at a heating rate of 1K/min under constant Argon flow of 20ml/min. The result confirms a 0.6% weight loss between 30 and 65°C, indicating evaporation of traces of the protective storage solvent dichloromethane.

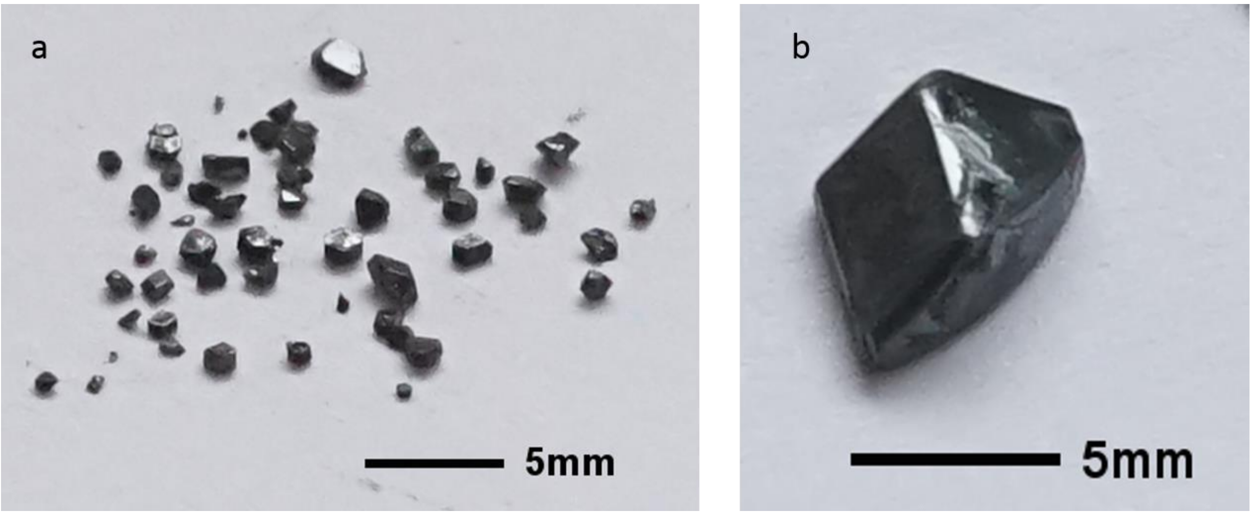

Figure S3 Photographs of freshly grown crystals a) within 20min and b) within 20 days

Figure S3 shows photographs of $CH_3NH_3PbI_3$ crystals freshly grown. The crystals in a) represent one typical batch grown from an equimolar precursor solution and were recovered after 20min from the solution while the large crystal in b) was grown over 20 days at slow heating of <1°C/h. The crystal in b) is strongly distorted in habit owing to the geometry of the growth setup which restricted the growth of some facets. All crystals show lustrous black surfaces and strong faceting, regardless of the growth time. Crystals <1mm diameter typically possess a regular habit and grow as dodecahedra or rhombo-hexagonal dodecahedra while large crystals typically exhibit strong distortion as result of preferential growth along favored facets. Optimized experimental geometry may enable growth of undistorted large crystals.
